# Review of Capacitive Touchscreen Technologies: Overview, Research Trends, and Machine Learning Approaches

**DOI:** 10.3390/s21144776

**Published:** 2021-07-13

**Authors:** Hyoungsik Nam, Ki-Hyuk Seol, Junhee Lee, Hyeonseong Cho, Sang Won Jung

**Affiliations:** Department of Information Display, Kyung Hee University, Seoul 02447, Korea; seol8118@khu.ac.kr (K.-H.S.); jklopp1007@khu.ac.kr (J.L.); chohs0713@khu.ac.kr (H.C.); mrswjung@khu.ac.kr (S.W.J.)

**Keywords:** touchscreen, capacitive, display, SNR, stylus, machine learning

## Abstract

Touchscreens have been studied and developed for a long time to provide user-friendly and intuitive interfaces on displays. This paper describes the touchscreen technologies in four categories of resistive, capacitive, acoustic wave, and optical methods. Then, it addresses the main studies of SNR improvement and stylus support on the capacitive touchscreens that have been widely adopted in most consumer electronics such as smartphones, tablet PCs, and notebook PCs. In addition, the machine learning approaches for capacitive touchscreens are explained in four applications of user identification/authentication, gesture detection, accuracy improvement, and input discrimination.

## 1. Introduction

Human beings collect a lot of information through their eyes, and many displays around us play a key role to transfer this visual information. Displays have evolved dramatically from cathode-ray tube (CRT) [1,2,3,4] via plasma display panel (PDP) [5,6,7,8,9,10] and liquid crystal display (LCD) [11,12,13,14,15] to cutting-edge organic light-emitting diode (OLED) [16,17,18,19,20,21,22] and micro-LED technologies [23,24,25,26,27,28]. This evolution has led to larger screen-size, slimmer design, lower weight, higher resolution, faster frame rate, brighter luminance, wider color gamut, longer life time, and lower power consumption in the large-size display applications such as monitors, televisions (TVs), and digital signage [29,30,31,32,33,34,35,36,37,38,39]. The resolutions of off-the-shelf displays have increased up to 8K (7680 × 4320) along with the high frame rate of 120 Hz and the larger screen sizes than 55-inch have taken more than 30% of overall TV set sales [40,41]. Even rollable OLED TVs were demonstrated in the consumer electronics show 2018 (CES2018) [42]. On the other side of the small-size display applications, higher density of pixels, narrower bezel, flexibility, bendability, rollability, and low power consumption have been achieved along with enhanced picture quality [43,44,45,46,47,48]. The latest smartphones contain the bezel-less screens of larger pixel densities than 450 pixel per inch (ppi) and smartphones with foldable displays are being sold on the market [49]. Recently, as augmented reality and virtual reality (AR/VR) attract substantial interest, the demand for high-performance near-eye displays is increasing further [50,51,52,53,54,55,56,57]. Consequently, the very high resolution OLED on silicon (OLEDoS) displays up to 4410 ppi have been reported [58,59,60,61,62,63].

On top of the role of a visual information provider, displays have supported the interaction with users by means of various user interfaces. Users can adjust the visual information on the screen by themselves. The very old but still popular representative user interfaces are mouse and keyboard [64,65,66]. There have also existed pen tablets for more elaborate works such as drawing and writing [67,68,69,70]. Because these devices work on the different planes separated from displays, additional markers such as cursors and pointers are needed. On the other hand, more intuitive input interfaces called touchscreens have been studied to directly interact with displays by touching displays [71,72,73,74]. Touchscreen technologies can be categorized into finger-touch and stylus-touch methods. While finger-touch methods include resistive, capacitive, acoustic wave, and optical approaches [75,76,77,78,79,80,81,82,83,84,85,86,87,88,89,90,91,92,93,94,95,96,97,98,99,100,101], stylus-touch ones cover up to electromagnetic resonance (EMR) schemes including finger-touch methodologies [102,103,104,105,106,107,108]. Recently, as wearable devices such as smartwatches and smartbands are becoming more popular, small-size displays are becoming further widespread with touch sensing functionality. However, because this very small-area screen cannot support multiple finger-touches and the whole area is covered even by a single finger, a variety of separate input modalities in the outside of the screen have been studied by using infrared (IR) line sensors, microphones, gaze trackers, IR proximity sensors, electric field sensors, deformation sensors, magnetic field sensors, and mechanical interfaces [109,110,111,112,113,114,115,116,117,118,119,120,121,122,123]. In addition, some approaches have coped with the limitation of the single touch by differentiating palm and finger or identifying pad, nail, tip, and knuckle of a finger [124,125]. Especially, because AR/VR displays are placed near to eyes, it is impossible to touch the screen directly. Therefore, other input tools using various sensors such as leap motion sensors, electromyograph sensors, inertial measurement units, eye-trackers, IR facial gesture sensors, cameras, and axis-tilt sensors, have been employed [126,127,128,129,130,131,132,133,134].

There have been also efforts to integrate machine learning (ML) approaches into touchscreen technologies. These ML networks are employed to add extra input tools, to improve the touch-sensing performance, to support the user identification/authentication, to discriminate finger-touches from others, and to capture the gestures [135,136,137,138,139,140,141,142,143,144,145,146,147,148,149,150,151,152,153,154,155,156,157,158,159,160,161,162,163,164].

There have been brief reviews of touchscreen technologies [76,96]. Walker [165] published many overview papers about a variety of touchscreen technologies from resistive to optical and electromagnetic resonance (EMR) stylus schemes. Those papers explained their histories, principles of operation, pros and cons, and applications. However, the technological details have not been handled such as algorithms, driving circuits, and ML approaches. Kwon et al. [166] reviewed capacitive touchscreen technologies including sensors, driving circuits, sensing methods, and stylus schemes in more detail. However, ML approaches were not introduced. Bello et al. [164] summarized ML approaches to improve security on touchscreen devices without addressing the touchscreen technologies. A variety of ML applications only for the security issues were addressed. This paper provides a unified and broader view of the touchscreen technologies with the detailed explanation and ML approaches in various scenarios.

The contributions of this paper are as follows:

- Providing the most comprehensive review about the touchscreen technologies. In particular, this describes various studies on sensing methods and ML approaches.
- Supplementing capacitive touchscreen techniques of the previous review paper [166] by focusing on research topics and results.
- Including various ML methods for user identification/authentication, gesture detection, accuracy improvement, and input discrimination.
- Proposing future directions for researches on touchscreen technologies integrated with ML networks.

This paper is organized as follows. Section 2 addresses the overview of the touchscreen technologies, and then Section 3 describes various studies on capacitive touchscreen applications that are integrated in most smartphone and notebook displays. Section 4 shows the ML approaches working with existing capacitive touchscreen technologies. Section 5 concludes this paper with some suggestions of the future directions.

## 2. Overview of Touchscreen Technologies

**Figure 1:**
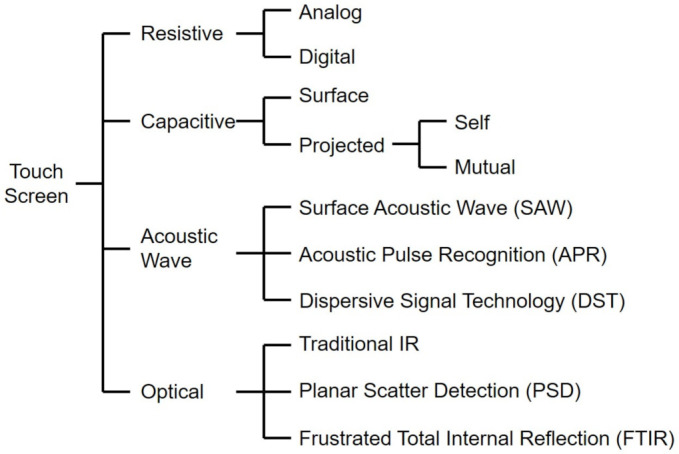
Categories of touchscreen technologies. This figure excludes the touchscreen techniques embedded in pixel areas in a display panel.

In this section, touchscreen technologies for finger as well as stylus have been simply addressed in terms of principles of operation, advantages, and drawbacks. We categorize the touchscreen technologies into four categories of resistive, capacitive, acoustic wave, and optical, and address further various techniques in each category as shown in Figure 1. Table 1 compares their specifications.

### 2.1. Resistive Touchscreen

**Figure 2:**
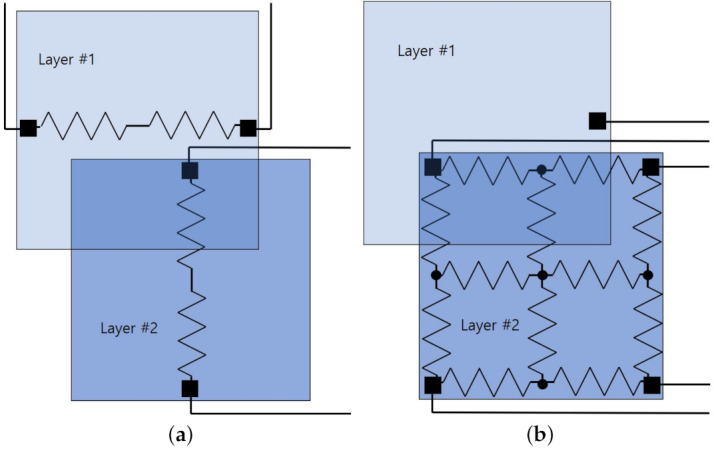
Two most popular resistive touchscreen architectures. (**a**) Four-wire. (**b**) Five-wire.

An analog resistive scheme is the oldest touchscreen technology [165]. It extracts touch coordinates by sampling the voltage at the touched area. The voltage is proportional to the location of the screen due to the voltage division based on the ratio of resistances from the current position to two opposite sides [78]. The most popular resistive touchscreen panels are fabricated by 4-wire and 5-wire architectures [79]. Both methods estimate x-axis and y-axis coordinates of a touch position sequentially. Normally, two separate layers are coated by the conductive films only at one side, and one layer should be composed of a flexible material. When the touch force is applied, the flexible layer is pressed to contact the other layer and to obtain the voltage at the contacted area. Four-wire structures use both layers to generate the voltage slopes as well as to sense the voltage as illustrated in Figure 2a. For example, after the flexible layer (Layer #1) generates the voltage slope at an x-axis and the other (Layer #2) senses the voltage, Layer #2 generates the voltage slope at an y-axis and Layer #1 senses the voltage. Five-wire ones apply voltages only to one specific layer (Layer #2) and use the other layer (Layer #1) only to sense the voltage as depicted in Figure 2b. Therefore, it is known that 5-wire schemes usually have a longer life time.

The advantages of the resistive touchscreen technology are to be able to work with anything, to be fabricated at the lowest cost, to be insensitive to any contaminants, and to consume low power. However, it has drawbacks of the only single touch support, the poor durability due to scratches, poking, and sharp objects, the poor optical clarity, and the relatively high touch force requirement [80,165].

On the other hand, there have been efforts to support multi-touch capability. Some researchers were trying to add the multi-touch functionality to a conventional structure by sensing the current consumption at voltage sources [167,168,169]. Whereas, other researchers divide the conductive films into multiple lines and columns that give rise to many separate overlapped areas [170,171,172], where each area can detect touches separately. This scheme is named as the digital resistive touchscreen [165]. Since the resistive touchscreen methods fall short of the capacitive schemes, the resistive touchscreen panels are being applied to the limited areas such as toys, office electronics, and card payment machines.

### 2.2. Capacitive Touchscreen

Capacitive touchscreens sense the change of the capacitance caused by the finger to estimate the touch position. While resistive schemes need the pressing force to make the actual contact between two conductive layers, capacitive methods can obtain the capacitance change just by the light touch on the screen. Consequently, it enables the smooth and fast scrolling, high durability, and excellent optical performance. In addition, any materials can be adopted for layers, for example, glasses and plastics, while resistive technologies require one flexible layer at least. Because the parasitic capacitance added by fingers is very small, large-size capacitive touchscreen panels are very difficult to implement and contaminants such as water and dusts can be also recognized as touches. Recently, the large size capacitive touchscreens have been reported based on the metal mesh structure [108,173]. It can support only capacitive input tools including fingers to make parasitic capacitors with electrodes of the touchscreen panel.

**Figure 3:**
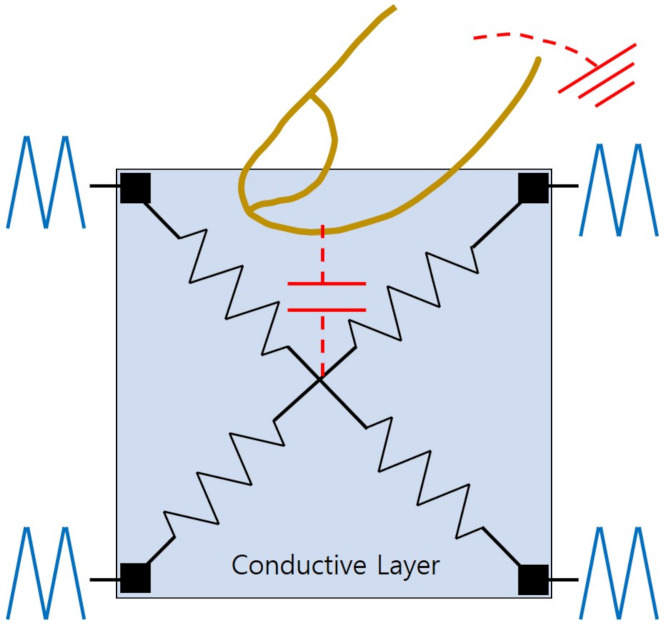
Surface-capacitive touchscreen. The touch location can be estimated from the current variation at four corner AC voltage sources caused by the finger touch.

The capacitive scheme is divided into surface-capacitive [81,83] and projected-capacitive methods [82,84]. Surface-capacitive touchscreens consist of one conductive layer of which four corners are connected to four perfectly synchronized alternative current (AC) voltage signals as described in Figure 3. While any difference does not occur without touches at these voltage sources, the finger touching the screen brings out the current difference in four voltage sources. As the voltage source is located nearer to the touch point, the current variation becomes larger due to the smaller resistive load. As a result, the touch locations are extracted from the ratio of the currents over four voltage sources. Even though it cannot deal with multiple touches at the same time, its high durability enables the integration in automated teller machines (ATMs).

The projected-capacitive methods can be further divided into self-capacitance and mutual capacitance architectures. Especially, the mutual capacitance has been the mainstream technology used in most consumer electronics such as smartphones, tablet PCs, and notebook PCs since the appearance of iPhones in 2007, because it can support multi-touch functions along with high durability and good optical clarity.

**Figure 4:**
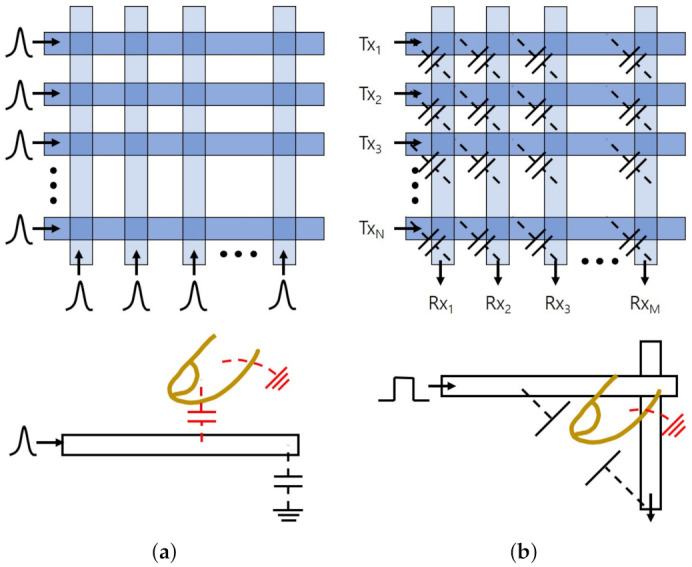
Projected-capacitive methods. (**a**) Self-capacitance. (**b**) Mutual capacitance.

In general, the projected-capacitive touchscreen panels use two patterned conductive layers that are separated and crossed to each other in the shape of a matrix. Horizontal and vertical patterns correspond to the position information of the touch event. While the self-capacitance senses the capacitance between layers and ground as shown in Figure 4a, the mutual capacitance measures the capacitance at the overlapped areas of horizontal and vertical patterns as presented in Figure 4b. Consequently, the finger touch increases the self-capacitance due to the additional parasitic capacitor in parallel and decreases the mutual capacitance due to the electric field loss by the finger placed between two electrodes.

The self-capacitance estimates x-axis and y-axis coordinates sequentially by measuring the capacitance of vertical and horizontal electrodes over the ground, respectively. Consequently, the multiple touches may cause ghost touches. For example, when there are two touches at locations of (x1, y1) and (x2, y2), the self-capacitance can figure out that there are touches at x1, x2, y1, and y2, separately, and then it provides two correct locations of (x1, y1) and (x2, y2) along with two additional ghost locations of (x1, y2) and (x2, y1) by four possible combinations of two x-axis data and two y-axis data. Thus, the self-capacitance has difficulty to support multi-touch functionality. To cope with this ghost touch issue, some panel makers use separate self-capacitance cells directly connected to the touchscreen controller that senses each capacitance variation, respectively, [174]. This approach has been implemented in the off-the-shelf smartphones.

On the other hand, because the mutual capacitance measures the overlap capacitance separately between vertical and horizontal conductive patterns, it can support multi-touch functions without any limits on the number of fingers. Therefore, it has become the widely used touchscreen technology today. The excitation pulses are applied to horizontal patterns and the transferred charges are measured through charge amplifiers at the ends of the vertical patterns. Since the amount of transferred charges is proportional to the mutual capacitance, the variation of capacitance can be detected. Section 3 will address the mutual capacitance approaches in more details.

While additional touchscreen panels on the displays require further electronics, the embedded touchscreen solutions that are called an in-cell touch can merge panel and touchscreen electronics into a single driver integrated circuit. Therefore, various in-cell approaches have been developed including self-capacitance cells and capacitive sensors embedded in pixel areas [174,175,176,177,178,179].

### 2.3. Acoustic Wave Touchscreen

**Figure 5:**
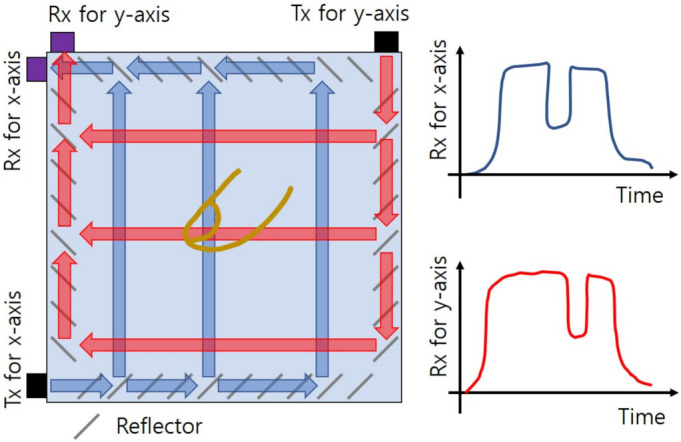
Surface acoustic wave (SAW) touchscreen. The ultrasonic waves move through multiple horizontal and vertical paths. The finger touch attenuates the received signal strength in the contacted wave paths and their delay information is converted to the touch locations.

The acoustic wave scheme is composed of a wave guide, sound wave sources, and receivers. The well-known technology is a surface acoustic wave (SAW) touchscreen as depicted in Figure 5 [85,86,87,91]. The SAW contains two pairs of ultrasonic transmitters and receivers to calculate x-axis and y-axis coordinates of touch locations, respectively. The reflectors in the bezel area generate multiple horizontal and vertical acoustic wave paths that have different arrival times at receivers. When a finger is placed in a certain path, the signals attenuated by that touch arrive at the receiver with corresponding delays that are converted into the position coordinates. Because the SAW needs only one wave guide layer, it has the most excellent optical performance. In addition, large size touchscreen and high durability are achievable. However, its sequential estimation of x-axis and y-axis coordinates gives rise to the same ghost touches as the self-capacitance method. It can also detect some input tools of soft materials to absorb waves and the sensing performance is sensitive to contaminants on the screen.

The another one is a bending wave scheme, where the sound wave caused by tapping on the screen is used as the sound source as well as the touch signal [88,89,90]. There are two methods of acoustic pulse recognition (APR) and dispersive signal technology (DST) [180]. The APR senses the bending waves by multiple piezoelectric transducers and processes them with the data stored in the memory to extract the touch positions. Therefore, the APR needs a prior process to sample and store the large amount of bending wave data at enough number of positions over the screen. However, because the bending wave characteristics are not deterministic, the resultant coordinates have some variance, leading to errors on the location estimation. Furthermore, the enough bending wave strength is required for the sensors to detect. The bending wave characteristics are dependent of the mounting structure and material. Since too large an amount of data is necessary for multi-touch cases, it supports only a single-touch input.

To cope with the requirement of the prior process to store the bending wave data in the APR, the DST extracts touch locations directly only from the measured bending wave data. Because the signal delay is affected by its frequency, the measured time and frequency information is used to reconstruct the bending wave pattern on the screen, which is converted to the touch coordinates. However, it also has several drawbacks such as only single touch support, high tapping strength, measurement variance, mounting dependency, as well as high computational power. In addition, both APR and DST cannot support the holding function because only the tapping action generates the sound waves.

### 2.4. Optical Touchscreen

The optical touchscreens are developed based on the invisible infrared (IR). The traditional IR-based touchscreen places transmitters at two sides and receivers at their opposite sides without any additional layers. Because the touches block the light path over the screen between a pair of transmitter and receiver, x-axis and y-axis coordinates can be obtained by finding the receivers’ positions that do not receive IR. While large-size displays and excellent optical clarity can be supported, the bezel needs some height over the screen for IR transmitters and receivers and the multiple touches cause the ghost touch issue.

**Figure 6:**
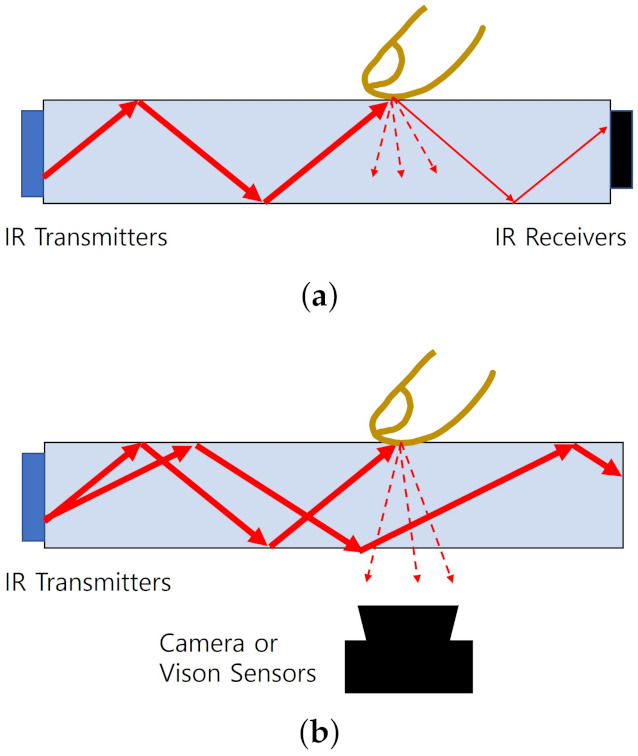
TIR-based IR touchscreen technologies. (**a**) PSD. (**b**) FTIR.

The other IR-based schemes such as planar scatter detection (PSD) [100,101] and frustrated total internal reflection (FTIR) [75,95,98,99] are similar to the acoustic wave approaches except for the use of IR instead of the sound wave. In the PSD, while the transmitters send the IR lights through the wave guide at the total internal reflection (TIR) condition, receivers sense them. When any touches are applied on the wave guide plate, it breaks the TIR condition out, therefore, the scattered and remaining TIR lights arrive at multiple receivers as described in Figure 6a, leading to the extraction of the touch location by the complex analysis. The PSD can support multi-touch and high image clarity, but the larger-size touchscreens require higher computational power to extract the touch location. The FTIR also makes use of the TIR condition, but the touch location is attained from the lights escaped toward the opposite plane to the touched one as depicted in Figure 6b. Those lights are captured by the external camera or vision sensors and the resultant images provide the information of touch locations. There also exist the embedded LCD solutions, where IR transmitters are allocated in the backlight and the vision sensors are placed in the pixel areas.

## 3. Main Research Trends in Mutual Capacitance Capacitive Touchscreen Technologies

As explained in the previous section, there have exist various touchscreen technologies by means of resistance, capacitance, sound wave, and IR. Among them, the capacitive touchscreen has become a mainstream scheme, especially, the mutual capacitance touchscreen is the most widely used technology on many consumer electronics such as smartphones, notebook PCs, tablet PCs, and smartwatches, because of its multi-touch support, slim form factor, high optical quality, excellent durability, smooth scrolling, and so on. Particularly, this section addresses the mutual capacitance capacitive touchscreens in more details. Unlike the self-capacitance method where the parasitic capacitor of a finger touch is connected to the self-capacitor in parallel, the mutual capacitance scheme experiences the capacitance reduced by electric field leakages into a finger. As a result, the touch location can be found out by searching the position which mutual capacitance is reduced.

**Figure 7:**
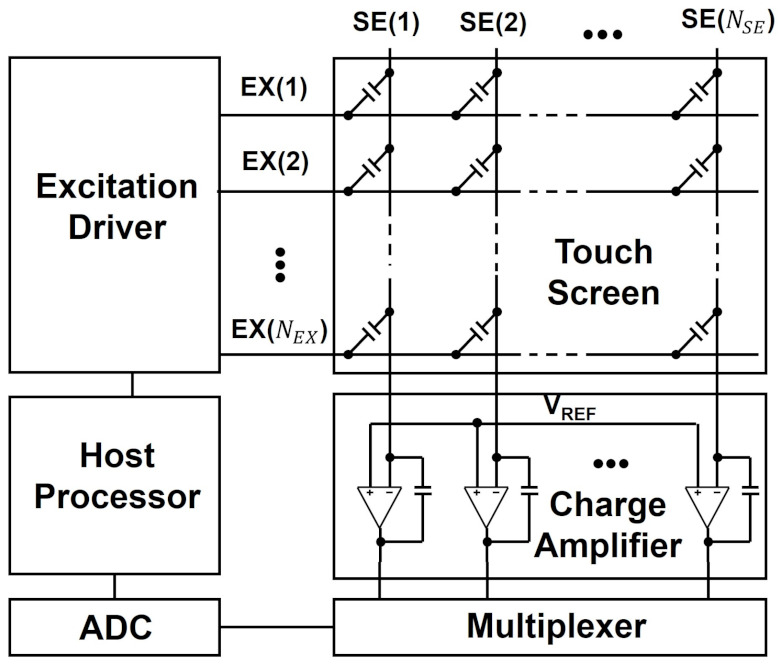
Block diagram of a conventional mutual capacitance capacitive touchscreen system. *N_EX_* and *N_SE_* are the numbers of excitation and sensing electrodes, respectively. The touch locations are estimated from the mutual capacitor array formed at the intersection areas of EX and SE lines.

**Figure 8:**
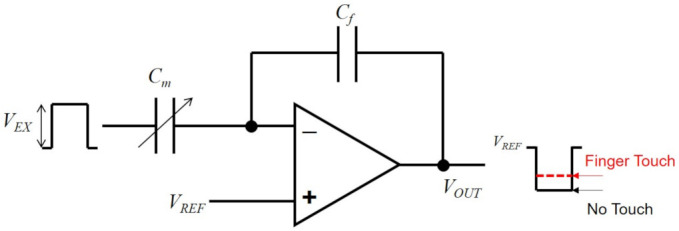
Charge amplifier circuit. The change of *C_m_* causes different output voltage levels (*V_OUT_*).

As shown in Figure 7, a conventional mutual capacitance capacitive touchscreen panel is composed of excitation (EX) electrodes and sensing (SE) electrodes, which give rise to the mutual capacitor array at their intersection areas [181,182]. Excitation drivers generate EX pulses sequentially in the way of line-by-line that arrive at charge amplifiers attached to SE lines through mutual capacitors. The non-inverting input terminals of these charge amplifiers are connected to the reference voltage ($V_{REF}$) and the charge transferred through a mutual capacitor ($C_{m}$) is converted through a feedback capacitor ($C_{f}$) into analog voltages ($V_{OUT}$) that are proportional to the mutual capacitance as presented in Equation (1). $V_{EX}$ is the amplitude of the EX pulse. When a user touches on the screen with a finger, the reduction on the mutual capacitance is sensed as the different output voltage of the charge amplifier from the voltage level obtained without any touches as illustrated in Figure 8. To improve the precision of the touch detection, the transferred charge is accumulated at the charge amplifiers over multiple EX pulses. In addition, a multiplexer (MUX) allows one analog-to-digital converter (ADC) to sample the output voltages of charge amplifiers in all SE lines sequentially. Finally, a host processor handles the digital data to determine the touch locations and it also controls excitation drivers.

(1) $$V_{OUT}=V_{REF}-\frac{C_{m}}{C_{f}}V_{EX}.$$

The mainstream studies in mutual capacitance schemes are (a) improving signal-to-noise ratio (SNR) to achieve higher accuracy as well as robustness over the noises and (b) utilizing additional input tools such as styli besides fingers. In addition, it is another research trend to integrate the pressure-sensing capability. However, the most approaches support this pressure sensing function through additional sensors [183,184], the separation distance changes [185,186], or the internal circuit of the stylus [108,187]. Because additional sensors and separation distance changes are out of this review’s scope, the stylus technologies are addressed along with their pressure sensing schemes.

### 3.1. SNR Improvement

**Figure 9:**
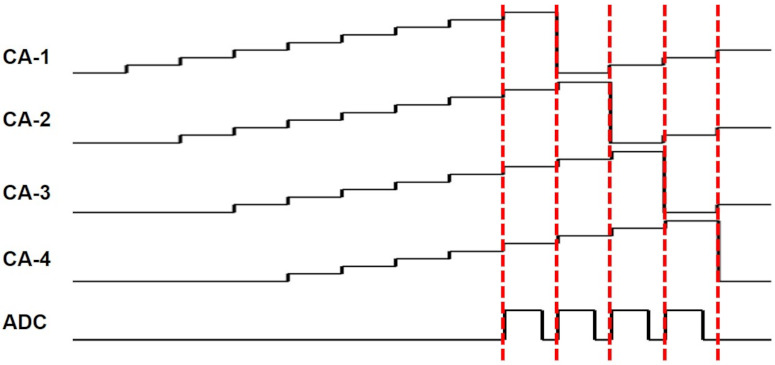
The SNR improvement is achieved by accumulating output pulses of charge amplifiers. CA-1 to CA-4 are the accumulated voltages for the outputs of four charge amplifiers. The outputs accumulated over several pulses are sampled by the ADC.

For the SNR improvement, various noises such as display noises, charger noises, and hum noises need to be addressed. Usually, the most sensing circuits employ the voltage accumulation at the output of the charge amplifier as shown in Figure 9, to suppress the noise power over the main signal, based on the assumption that the noise is independent and identically distributed [188]. Because the noise power and the signal power are proportional to the number of pulses and its squared value, respectively, the SNR improvement is achieved.

**Figure 10:**
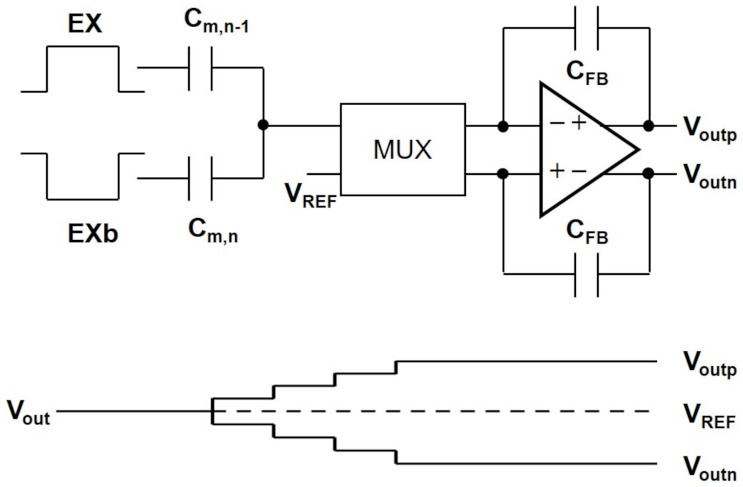
The dynamic range of the output voltage is enhanced by using differential-ended charge amplifier and out-of-phase excitation pulses.

Yang et al. [189] employed the differential-ended charge amplifier with two out-of-phase excitation pulses (EX, EXb) as depicted in Figure 10. With the single-ended amplifier, the output dynamic range (DR) is limited by the default mutual capacitance without touches. However, the proposed differential structure reflected only the difference of the mutual capacitance ($C_{m}$) over the adjacent line’s capacitor on the output voltages. In addition, the differential-ended amplifier gave rise to the doubled DR by non-inverting and inverting outputs ($V_{outp}$, $V_{outn}$). Therefore, the increased signal power led to the improved SNR performance.

Kim et al. [190] proposed the common-mode noise cancellation by subtracting the signals of the adjacent EX lines. Since the parasitic capacitance between neighboring EX lines of a touchscreen panel and display panel are almost equal, the injected noises from the display to the touchscreen would be similar, therefore, the differential sensing method over EX pulses of two neighboring lines could eliminate the common-mode display noise. Yang et al. [191] added a charge-interpolation filtered-delta-integration (CI-FDI) scheme to cancel the charger noise. The large noise is detected, and then the noise is prevented by the charge-interpolation.

**Figure 11:**
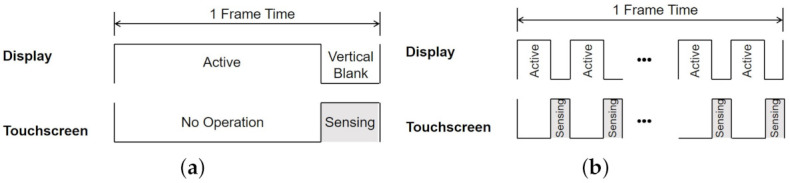
Touch sensing methods during the vertical blank periods. (**a**) Sensing during one vertical blank period. (**b**) TDMS sensing during divided vertical blank periods in the middle of a frame.

As the other method to reduce the display noise components, it was proposed that the sensing operation was conducted only during the vertical blank interval as presented in Figure 11a. However, the sensing operation over the whole touchscreen area should be finished within very short period of time at the end of a frame time. Since it could not support smooth scrolling motions, the time division multiple sensing (TDMS) was introduced to spread the touch sensing functions evenly over a frame time [174,192], where the divided vertical blank parts were added in the middle of the active interval by stopping the scanning operations as illustrated in Figure 11b.

Miura et al. [193] adopted a two-step dual-mode scheme that performed self- as well as mutual-capacitance measurements. After the self-capacitance measurement found the touched areas, the mutual-capacitance measurement provided the fine touch location over the touched areas. Therefore, it achieved the high resolution of 80 × 80 and the high scan rate of 322 Hz.

**Figure 12:**
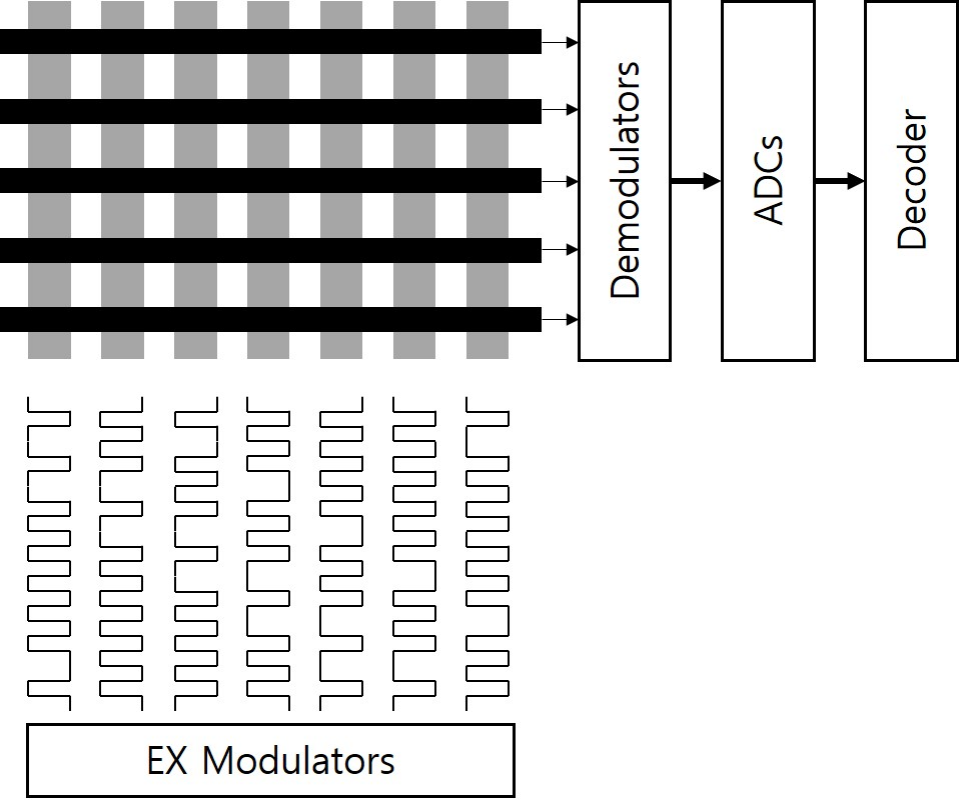
Code division multiple sensing. CDMS enables the multiple capacitance sensing at the same time by transmitting orthogonal codes through multiple EX lines. The charge amplifiers’ outputs are demodulated and converted into digital data that are decoded as multiple simultaneous touch locations.

An output accumulation sensing method can improve the SNR by applying multiple EX pulses per one touch position estimation, but lowers the scan rate inevitably. To improve SNR as well as scan rate simultaneously, Shin et al. [194] implemented a code-division multiple-sensing (CDMS) method. While multiple EX pulses per one EX line are used for sensing one touch position like the output accumulation sensing method, multiple EX electrodes are excited with orthogonal patterns simultaneously as illustrated in Figure 12. Then, the touch information over multiple positions was obtained at the same time through the decoding process. As a result, the CDMS method achieves a much higher scan rate without the SNR degradation [195,196].

**Figure 13:**
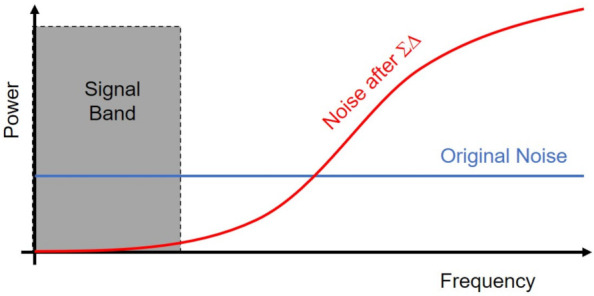
The sigma delta modulator shapes the noise power by moving the low frequency noises to the high frequency region. Therefore, the SNR at the signal band is improved by means of low-pass filtering.

Park et al. [197] used the delta-sigma modulator in the ADC to move the low frequency noise to the high frequency region as shown in Figure 13. By applying a low-pass filter to remove the high frequency noise components, the noise power within the touch signal band was substantially reduced, leading to the improved SNR performance.

An et al. [108] introduced the multiple-frequency driving scheme. A fast Fourier transform (FFT) was applied to find the touch locations because EX pulses had different frequencies for each EX line, that is, the number of EX frequencies is equal to the number of EX lines. Furthermore, a spectrum of external noises was acquired, and then the frequencies of EX signals were located in the low noise region for the high SNR. However, in order to implement the FFT functionality, EX signals must be driven at a very high frequency for the large number of EX lines, which leads to the increased power consumption and hardware complexity. In addition, An et al. [198] integrated the amplitude-modulation to the multiple-frequency driving scheme to reduce the charge-overflow. The excitation pulses were amplitude-modulated to reduce their amplitudes with the same periodicity. It achieved 33.9 dB charge-overflow reduction, leading to the high SNR performance.

The above SNR improving technologies are summarized in Table 2 including SNR, scan rate, touchscreen resolution, year, and reference.

### 3.2. Stylus Support

**Figure 14:**
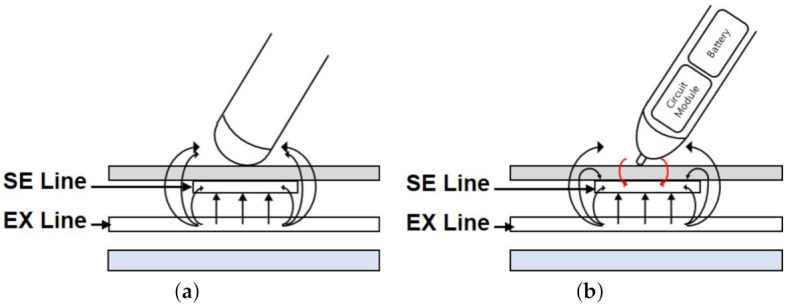
Stylus technologies for capacitive touchscreens. (**a**) Passive stylus. (**b**) Active stylus.

Handwriting and drawing applications on touchscreens require more elaborate input tools than a finger. The representative input tool is a stylus that has the shape of a pen. The simplest stylus implementation called a passive stylus is based on the conductive tip that imitates a finger touch as depicted in Figure 14a [103]. However, since the contact area must be large enough to be comparable to that of a finger, it is not adequate to elaborate works. In addition, it cannot support smooth scrolling and high durability due to its rubber-type tip. Additionally, the passive stylus is not distinguished from a finger. Lin et al. [199] showed that the pressure level could be sensed in the passive stylus due to the contact area change of the deformable head proportional to the pressing force.

Active stylus schemes have been introduced to support elaborate works even with small-sized tips as illustrated in Figure 14b. The early active stylus [105] senses EX pulses from the touchscreen and transmits its inverted and boosted pulses back to the touchscreen via a tip. As these inverted pulses reduce the amount of charges transferred to the charge amplifiers, the resultant outputs of charge amplifiers become equivalent to the voltage levels caused by the reduced mutual capacitance. The process of amplifying the sensed EX signal allows for the use of much smaller radius tips than the passive stylus.

If larger inverted pulses are applied to the tip, the different output voltage from finger-touch as well as no-touch will be obtained at charge amplifiers, which enables the stylus differentiation from the finger [106]. However, this scheme needs much higher voltage amplification at stylus circuits than EX pulses of the touchscreen to give rise to the additional voltage level required for the stylus discrimination. It reduces both dynamic range and SNR for the finger-touch detection when the ADC has the fixed input voltage range. In addition, the boosted voltage levels should be separately adjusted in accordance with touchscreens.

**Figure 15:**
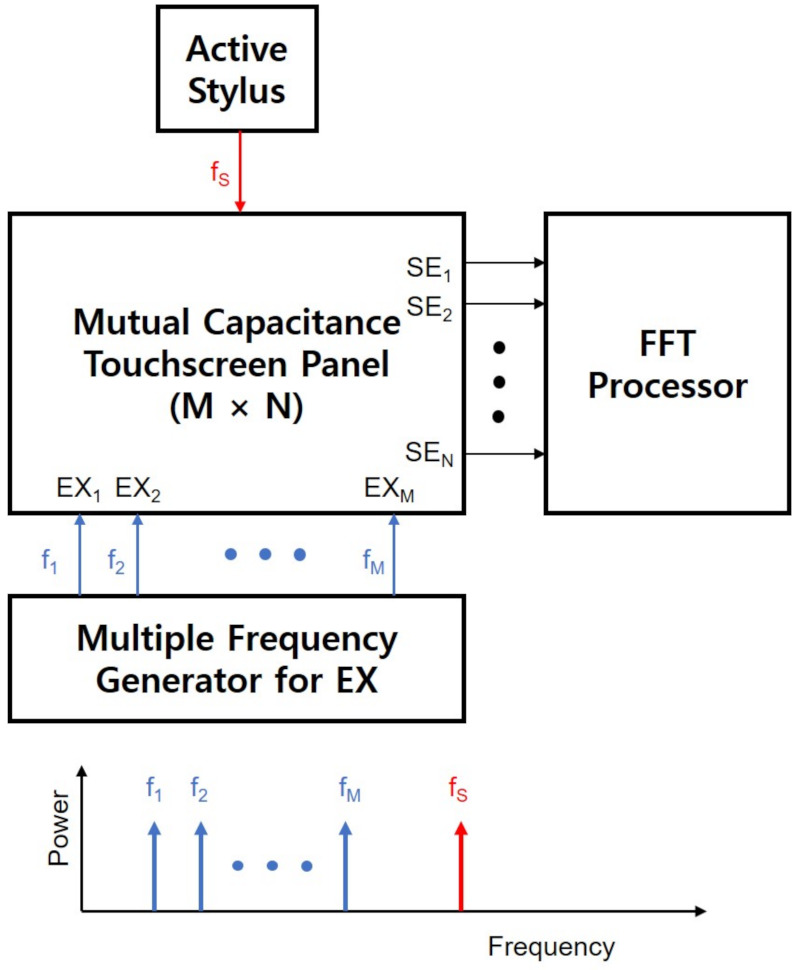
Multiple-frequency driving touchscreen scheme based on an FFT processor. EX lines as well as stylus can be discriminated in the frequency domain at the same time by assigning different frequencies.

An et al. [108] introduced the other active stylus based on the multiple-frequency driving scheme as presented in Figure 15. It could distinguish the stylus from the finger, because tip pulses of the stylus had different frequencies from EX pulses of the touchscreen. Even though it can achieve the high SNR, it required the high computational power due to the FFT implementation. Its stylus supported pressure and tilt sensing functions by means of additional force gauge and gyro sensor.

**Figure 16:**
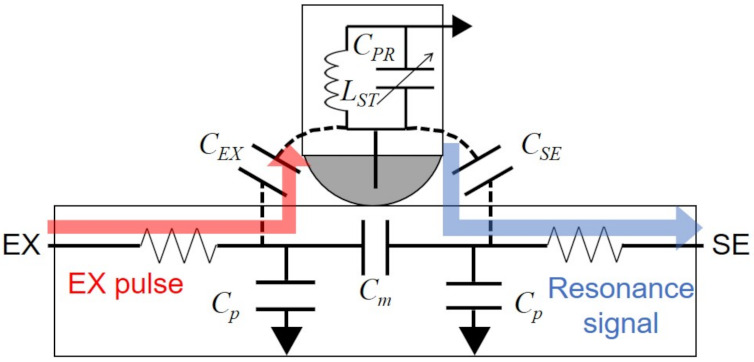
Electrically coupled resonance stylus. EX pulses are transmitted to the inside LC circuit and resonance signals are transferred to the coupled SE line. *C_m_* is the mutual capacitance, *C_p_* is the parasitic capacitor of EX and SE lines, *C_EX_* is the coupling capacitance between EX line and stylus, and *C_SE_* is the coupling capacitance between SE line and stylus. *L_ST_* and *C_PR_* are inductance and capacitance of the resonance circuit in the stylus.

Lee et al. [187] proposed an electrically coupled resonance (ECR) stylus. As depicted in Figure 16, when the excitation pulse is asserted, the pulse is transferred to an LC resonance circuit of inductor ($L_{ST}$) and capacitor ($C_{PR}$) in the ECR stylus and the signal of the resonance frequency is transmitted from the tip of the stylus to the touch sensing circuits. Since the ECR stylus consists of only passive elements, it does not need any batteries. In addition, the finger touch can be differentiated from the stylus. Because $C_{PR}$ is modified by the pressure, the pressure level can be differentiated by the resonance frequency. However, the large excitation pulse amplitude is necessary to generate the resonance pulse signals that can be detected at the touch sensor circuits. Besides, the frequency detection requires the increased hardware complexity.

**Figure 17:**
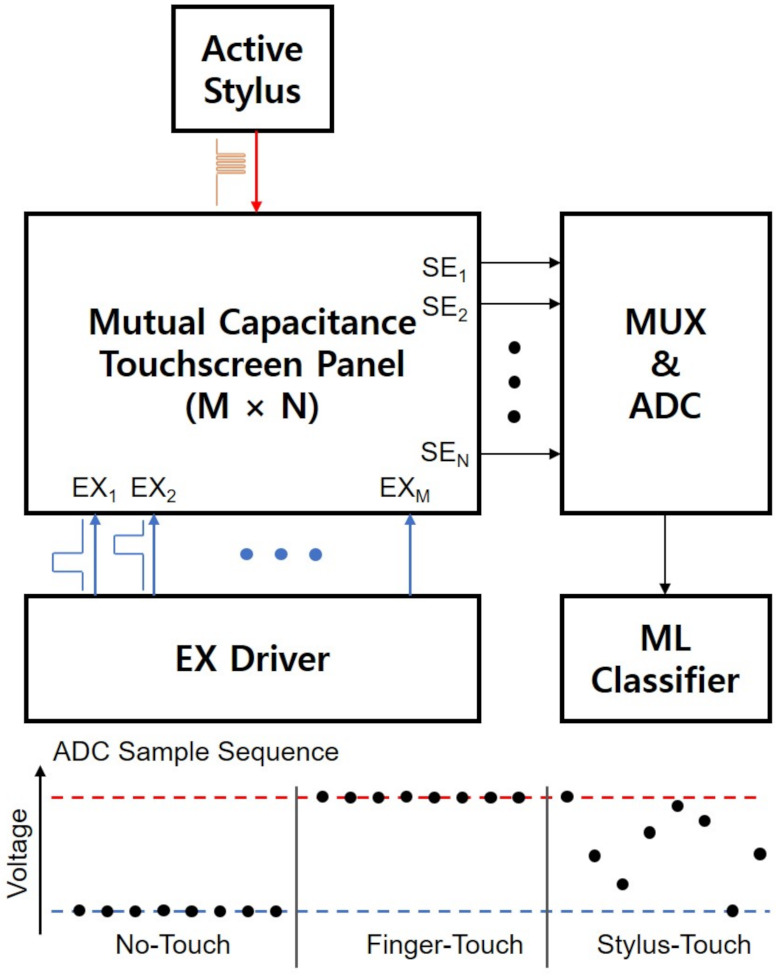
ML-based touchscreen scheme. The higher frequency pulses of a stylus generate the different sequence from finger-touch and no-touch, and three touches are discriminated by ML-based classifiers.

Seol et al. [157,163] adopted the active stylus scheme that used the higher frequency pulses to generate different patterns from EX pulses for the finger-touch detection. It discriminated stylus-touches from no-touches and finger-touches without the high complicated FFT functionality by machine-learning-based classifiers as depicted in Figure 17. It also showed that the proposed method allowed for the effective data communication between touchscreens and styli. On the other hand, since the patterns of stylus-touches were placed in between finger-touches and no-touches, the achieved SNR was relatively low compared to the state-of-the-art high SNR touchscreen technologies.

While previous passive and active ways are grounded in the capacitive touchscreen panel, there is a stylus with a different approach that works with an electro-magnetic resonance (EMR) technology [107]. Since EMR responds only to the stylus and the capacitive touchscreen panel senses only the fingers, two separate touch sensing schemes enable the finger to be distinguished from the stylus. However, this technique needs additional layers that increase hardware complexity as well as manufacturing cost.

The stylus technologies that can be implemented on the capacitive touchscreen are summarized in Table 3 in terms of tip size, stylus discrimination support, SNR degradation, computational cost, and hardware complexity.

## 4. Machine Learning Approaches in Mutual Capacitance Capacitive Touchscreen

Several ML algorithms have been employed to the capacitive touchscreen in a variety of applications such as user identification/authentication, gesture detection, accuracy improvement, and input discrimination. While many approaches used traditional ML techniques of decision tree (DT), random forest (RF), naive Bayes (NB), radial basis function network (RBFN), back propagation neural network (BPNN), support vector machine (SVM), and Gaussian process regression (GPR), the latest ML networks, such as convolutional neural network (CNN), anomaly detection (AD), and recurrent neural network (RNN), have been also utilized in the touchscreen field. The following machine learning applications are implemented on the off-the-shelf smartphones and tablet PCs with mutual capacitance capacitive touchscreens.

### 4.1. User Identification/Authentication

The user identification and authentication is the most active field to apply the machine learning to the touchscreen because the touch behaviors are different according to the users. Kolly et al. [135] used DT, RF, and NB classifiers over touch gestures for the user authentication application. Mean and maximal pressures, the point in the time at the maximal pressure event, minimal and maximal gradients of the pressure, the hold time, mean x-axis and y-axis positions, and the variances in x-axis and y-axis directions were employed as input features that were crowdsourced by designing a quiz game. The resultant identification accuracy for five users was 80% or more. It also proposed the anomaly detection for the user authentication based on the learned distribution of features from 5 button touch events. The equal error rate of false reject ratio (FRR) and false accept ratio (FAR) was estimated as about 30%.

Feng et al. [138] proposed a finger-gesture authentication system using touchscreen (FAST). FAST collected touch gesture information including gesture type, x-axis and y-axis coordinates, directions of the finger motion, finger motion speed, pressure, and the distance between multi-touch points. Totally 53 features for each gesture and six gestures of down to up swipe, up to down swipe, left to right swipe, right to left swipe, zoom-in and zoom-out were put into DT, RF, and NB classifiers. It achieved FAR of 4.66% and FRR of 0.13% for the continuous post-login user authentication.

For the user authentication, Meng et al. [139] constructed 21 features such as average touch movement speeds for eight directions, fractions of touch movements for eight directions, average single-touch time, average multi-touch time, number of touch movements per session, number of single-touch events per session, and number of multi-touch events per session. They evaluated the performance of DT, NB, Kstar, RBFN, and BPNN, leading to the conclusion that RBFN showed the best performance with FAR and FRR of 7.08% and 8.34%. In addition, the particle swarm optimization (PSO) with a RBFN classifier reduced FAR and FRR further to 2.5% and 3.34%, respectively.

Saravanan et al. [140] proposed the authentication scheme based on the user’s touch interaction with common user interface elements such as buttons, checkboxes, and sliders. Using SVM and RF, they achieved average accuracies of 97.9% and 96.79% with mobile phone and tablet PC, respectively.

Guo et al. [143] proposed CapAuth that is a user identification and authentication technique based on capacitive touchscreen data combined with machine learning classifiers. It used the capacitive image of the hands-flat pose revealing the more distinguishing features. CapAuth was built based on quadratic-kernel SVM classifiers, a binary classifier for authentication and a multi-class one-to-one classifier for identification. The measure FRR and FAR for authentication were 5.5% and 0.1%, respectively. The accuracy of the identification was 94.0% for 20 users.

Rilvan et al. [148] used four fingers, thumb, as well as ear as the types of biometrics for authentication with the machine learning classifiers such as SVM and RF. It achieved the maximum authentication accuracy of 98.84% over four fingers with SVM and maximum identification accuracy of 97.61% by four fingers with RF.

Meng et al. [152] enhanced touch behavioral authentication by cost-based classifier selection, where the best classifier with the lowest cost value was selected among a set of classifiers including DT, NB, RBFN, and BPNN. It had nine touch features such as the number of touch movements per session, the number of single-touch events per session, the number of multi-touch events per session, the average time duration of touch movements per session, the average time duration of single-touch per session, the average time duration of multi-touch per session, average speed of touch movement, average touch size, and average touch pressure. The average error rate of FAR and FRR was measured to be less than 5% for 15 sessions or more.

### 4.2. Gesture Detection

The user identification and authentication require various touch data such as location, speed, force, and gestures as features that are of the most importance for the better performance. Therefore, there have been researches to extract the gesture data from the touch data by means of machine learning algorithms. Xiao et al. [146] came up with an approach for estimating 3D finger angles such as pitch and yaw relative to a touchscreen’s surface. It used the capacitive image that was the capacitance measured at each point of a touch sensor’s capacitive grid. The pitch was estimated by a Gaussian process regression with 42 features and the yaw was computed by the major axis of the ellipsoid of the sensed touch pixels. While mean pitch errors were 9.7 and 14.5, mean yaw errors were 26.8 and 31.7 for phone and watch, respectively. It also proposed the possible applications such as zoom and rotate functions even with a single-touch event which would be very useful in small-size watch displays.

Mayer et al. [151] proposed the neural network approach to estimate the finger orientation of pitch and yaw. They evaluated the performance over separated deep neural networks (DNN) for pitch and yaw, combined DNN with two output neurons, and CNN along with L2 regularization [200,201] and batch normalization (BatchNorm) [202]. The blob detection provided the 15 × 22 sized data that was fed into neural networks. It achieved the best pitch error of 12.75 with CNN+L2+BatchNorm and the best yaw error of 17.6 with CNN.

Boceck et al. [161] extracted the pressure from capacitive images by using CNN. It used a ReLU function as an activation function, and dropout layers. Final fully connected layers contained LeakyReLU and L1/L2 regularization. They achieved the lower root mean square error of 471.99 g, compared to 583.36 g and 593.51 g of RF and SVM.

Schweigert et al. [162] added knuckle related features by differentiating knuckles from fingers and classified 17 finger and knuckle gestures by CNN and long short-term memory (LSTM). CNN layers extracted the representation from the 15 × 27 capacitive image and then LSTM layers generated 17 outputs over 50 consecutive images through a softmax activation function. Seventeen gestures contained tap, two tap, swipe left, swipe right, swipe up, swipe down, two swipe up, two swipe down, circle, arrowhead left, arrowhead right, rotate, and five additional gestures. It achieved the accuracies of 97.9% and 86.8% on train and test sets, respectively.

### 4.3. Accuracy Improvement

Weir et al. [137] adopted GPR to find a mapping between two-dimensional reported touch locations and a corresponding intended two-dimensional touch location on the display. It collected the touch data by randomly displaying the crosshairs that the users had to touch. The resultant average reductions in error rates were 23.79% for 2 mm buttons, 14.79% for 3 mm buttons, and 5.11% for 4 mm buttons.

Fischer et al. [155] presented a system using capacitive sensing to accurately classify hand touches and proximity. Touch data were collected through 50 finger touches with different fingers, angles, locations and speeds, 25 glove touches, and 20 non-valid touches. Then, the collected data were further processed by dimensionality reduction, data augmentation, and normalization. Hidden Markov model (HMM) and RNN of gated recurrent units (GRUs) were evaluated as the classifier over three classes of non-touch, near, and touch. The RNN model showed the better overall accuracy of 97.1% even with gloves while HMM achieved the accuracy performance of 84.21%.

Kumar et al. [158] improved the accuracy of touch locations by CNN. The dataset consisted of capacitive images at the dimension of 15 × 27 as well as the estimated touch positions represented by the centroid of the touch blob. The proposed CNN achieved an average error offset of 41.23 pixels based on a screen resolution of 1920 × 1080 on a 4.94 inch display. It was the improvement of 23.0%, compared to the error offset of 50.7 pixels of the standard touch controller.

Kim et al. [160] introduced a sensor substitution system that generates time-series sensor data based on RNN. It estimated capacitive touch sensor signals by motion and audio signals caused by touch. By other types of multivariate time-series signals, the touch sensor sequences were supplemented even at dynamic and hostile environments that degraded the touch sensor’s performance.

### 4.4. Input Discrimination

There have been studies on the discrimination of inputs such as fingers, palm, and stylus. Schwarz et al. [142] employed the decision tree to distinguish between legitimate stylus and palm based on spatiotemporal touch features and iterative classification. It identified five properties of palms such as the large contact area, the segmentation into a collection of touch points, the clustering, the area change, and the little movements. As a result, min distance to other touches, number of touch events, and min/mean/max/stdev of touch radius were used as the features. The instant classification achieved an accuracy of 98.4% and the continuous iterative classification increased the accuracy to 99.5%.

Le et al. [154] differentiated between touches of fingers and palm to devise an additional input modality. Their PalmTouch showed possible one-handed and two-handed palm interactions by placing flat hand or fist on the screen. It used the capacitive images for the features and CNN for the classification, leading to the accuracy of 99.53%.

Le et al. [153] proposed the finger-aware interaction that identified fingers touching the whole device surface to add the input modalities. In a prototype, front and back side touchscreens were developed by two stacked smartphones and their three edges were attached with 37 capacitive sensors. It used CNN with L2 regularization to obtain 15 outputs that were a three-dimensional coordinate, (x, y, z), for five fingers. The identification accuracy was 95.78% with the position error of 0.74 cm.

Seol et al. [157,163,203] employed the machine learning based classifiers such as SVM and autoencoder-based AD for finger and stylus discrimination. The higher frequency pulses were transmitted from a stylus to a capacitive touchscreen and the outputs of the charge amplifiers were sampled by ADC and classified by the classifier into no-touch, finger-touch, and stylus-touch. While no-touch and finger-touch were the constant level sample sequences, the stylus-touch was the random sequence between two constant levels. Therefore, SVM and AD classifiers achieved lower bit error rates (BERs) than $10^{-6}$ with the palm rejection. In addition, it was shown that its data communication algorithm could be applied in data transmission and user identification.

## 5. Conclusions and Future Directions

In this paper, we have provided an extensive review on touchscreen technologies. We mainly dealt with the overview of various touchscreen schemes from resistive to optical methods, and two main research directions of SNR improvement and stylus support as well as machine learning approaches in mutual capacitance capacitive touchscreens that are the most widely adopted scheme at present in smartphones, tablet PCs, notebook PCs, and smartwatches. For the aspect of the SNR improvement, accumulation, differential sensing, TMDS, dual mode of self and mutual capacitance, CDMS, delta-sigma modulation, and multiple-frequency driving have been introduced. High SNRs have been achieved by reduced noises and increased dynamic ranges. For the stylus support, passive, active, multiple frequency driving, ECR, and ML-based schemes have been addressed along with their pressure sensing capabilities. The machine learning applications in capacitive touchscreens have been classified in four categories of user identification/authentication, gesture detection, accuracy improvement, and input discrimination by means of a variety of algorithms such as DT, RF, NB, RBFN, BPNN, SVM, GPR, CNN, AD, and RNN.

Although many advancements have been accomplished in touchscreen technologies, challenges still exist in various fields. We will point out some of these challenges. We hope that this review not only helps the understanding of the touchscreen technologies but also paves the way to future researches on integrating machine learning algorithms into touchscreens for more various applications. As the resolutions of touchscreens are getting larger, fingerprints can be detected on any locations of the screen without additional sensors [204,205]. However, high power consumption and low scan rate should be addressed. One of possible solutions would be the super-resolution (SR) that gives rise to the high resolution capacitive image from the low resolution capacitive image. Many deep-learning-based SR algorithms have been reported [206,207]. As discussed in the previous sections, the SNR is one of the most important performance metrics and the high SNR is required to integrate touch sensing and display driving electronics into one integrated circuit. There exists the research field of de-noising that generates the clean one from the noisy image. Therefore, this de-noising scheme can be applied to enhance the SNR over the acquired capacitive images. Lastly, because latest smartphones, tablet PCs, and notebook PCs contain many sensors such as cameras, IR sensors, microphones, accelerometers, and gyroscopes besides touchscreens, there will keep being many approaches based on multi-sensor fusion technologies in the user interface field.

## Figures and Tables

**Table 1:** Comparisons of touchscreen technologies.

Category	Resistive	Capacitive	Acoustic	Optical
# Layers	2	2	1	0 (Traditional)
				1 (PSD, FTIR)
Touch	High	Low	Average	High
Diversity				
Image Clarity	75–85%	85–90%	92–%	88–%
(Transmittance)				
Multi-	No (Analog)	No (Surface, Self)	No	No (Traditional)
Touch	Yes (Digital)	Yes (Mutual)		Yes (PSD, FTIR)
Durability	Poor	Good	Best	Best
Computational	Low	Average	High	Low (Traditional)
Power				High (PSD, FTIR)
Dimension	Small	Small (ITO)	Large	Large
		Large (Metal Mesh)		
Touch/Tap	Strong	Light	Average	Light
Force				
Resistance to	Best	Poor	Good	Good
Contaminants				
Holding	Yes	Yes	Yes (SAW)	Yes
Function			No (APR, DST)	
Mounting	No	No	No (SAW)	No
Dependency			Yes (APR, DST)	

**Table 2:** Specification comparison of capacitive touchscreen technologies.

Approach	SNR (dB)	Scan Rate (Hz)	Resolution	Year	Reference
Accumulation	24	65	20 × 16	2010	[188]
High DR	12.6	140	53 × 29	2011	[189]
Noise Cancel	35	120	30 SE	2012	[190]
CI-FDI	39	27	43 × 24	2013	[191]
CDMS	55	240	30 × 24	2013	[194]
	72		32 × 10	2016	[196]
Dual-mode	41	322	80 × 80	2015	[193]
TDMS	52	120	80 × 45	2015	[174]
Noise	40	6300	8 × 12	2014	[197]
Shaping	67	50			
Multiple	61	3900	64 × 104	2017	[108]
Frequency	61.6	2930		2020	[198]

**Table 3:** Comparison of stylus technologies for capacitive touchscreens.

Reference	Passive	Active	FFT	ML	EMR	ECR
	[103,199]	[105,106]		[157,163]		
Tip Size	Large	Small	Small	Small	Small	Small
Stylus	No	No ([105])	Yes	Yes	Yes	Yes
Discrimination		Yes ([106])				
SNR	No	No ([105])	No	Yes	No	Yes
Degradation		Yes ([106])				
Pressure	Yes [199]	-	Yes	No	Yes	Yes
Sensing						
Computational	Low	Low	High	Medium	Low	High
Cost						
Hardware	Low	Low	High	Low	High	High
Complexity						

## Data Availability

Data sharing not applicable.
